# MiR-29b Alleviates High Glucose-induced Inflammation and Apoptosis in Podocytes by Down-regulating PRKAB2

**DOI:** 10.2174/0118715303267375231204103200

**Published:** 2024-01-08

**Authors:** Hongxiu Du, Yakun Wang, Yingchun Zhu, Xiaoying Li, Tingying Zhu, Qianqian Wu, Fangfang Zha

**Affiliations:** 1 Department of Nephrology, Qingpu Branch of Zhongshan Hospital Affiliated to Fudan University, 1158 Gongyuan East Road, Qingpu District, Shanghai, 201799, China

**Keywords:** Cell apoptosis, diabetic nephropathy, inflammation, microRNAs, podocytes, PRKAB2 protein

## Abstract

**Background:**

Podocyte injury and inflammatory response are the core contributors to the pathogenesis of diabetic nephropathy. This study aims to identify novel regulatory miRNAs and elucidate their underlying mechanisms, which will help us understand the pathogenesis of diabetic nephropathy more comprehensively.

**Materials and Methods:**

Different glucose concentrations were used to treat podocytes to mimic the pathology of diabetic nephropathy *in vitro*. Flow cytometry was used to determine cell apoptosis. Inflammatory cytokines released by podocytes were measured by using an enzyme-linked immunosorbent assay (ELISA). Western Blot was used to detect the expression of PRKAB2 protein in podocytes.

**Results:**

Genecard and g: profiler results revealed that miR-29b might be involved in regulating HG-induced cell injury. QRT-PCR indicated that HG-induced downregulation of miR-29b in podocytes. MiR-29b knockdown promoted cell apoptosis and inflammatory response in podocytes. MiR-29b overexpression repressed cell apoptosis and inflammatory response induced by high glucose treatment in podocytes. Luciferase reporter assay and Western Blot showed that miR-29b targeted PRKAB2 to negatively regulate PRKAB2 expression directly. Knockdown of PRKAB2 reversed the increased cell apoptosis and inflammation induced by miR-29b inhibitors.

**Conclusion:**

MiR-29b plays a role in inhibiting inflammation and apoptosis in high glucose (HG) treated podocytes by negatively regulating PRKAB2 expression. This study provides new potential targets and ideas for the treatment of diabetic nephropathy.

## 1 INTRODUCTION

End-stage renal disease (ESRD) is one of the most intractable diseases of clinical kidney disease worldwide [1]. Diabetes can significantly increase the risk of CKD (chronic kidney disease) and ESRD [2]. Diabetic nephropathy is diagnosed when the diabetic patient gets the symptoms of microalbuminuria [3]. Podocytes are highly differentiated glomerular epithelial cells, which are essential in maintaining the structure and function of the glomerular filtration barrier [4]. Podocyte injury is the leading cause of diabetic kidney disease [5]. The imbalance of metabolism homeostasis in the renal podocyte region leads to the occurrence and development of diabetic nephropathy, making it a research hotspot in this field [6]. In the diabetic hyperglycemic environment, the level of inflammation in the body is significantly increased, and the increase of the level of inflammatory factors can induce podocyte injury and proteinuria and finally show diabetic nephropathy [7, 8].

Inflammatory pathways have been considered an essential part of the pathogenesis and development of diabetic nephropathy, and the identification of new inflammatory targets may benefit the development of novel therapies [9]. Many molecules related to the inflammatory pathway in diabetic nephropathy may be the potential target or biomarker for the treatment and diagnosis of diabetic nephropathies, such as inflammation-related receptors, kinases, and cytokines [10]. Understanding these molecular pathways may contribute to the transformation of the research achievements into anti-inflammatory treatment strategies.

MicroRNAs (miRNAs) are an essential kind of small RNAs that play numerous physiological roles by targeting the gene translation process through binding to the 3’ untranslated region (3' UTR) of the genes [11]. MiRNAs participate in many physiological and pathological processes, such as cell metabolism, differentiation, proliferation, and diabetic nephropathy [12]. Thus, miRNAs are potential targets and biomarkers in diabetic nephropathy [13]. Furthermore, many miRNAs are involved in the occurrence and development of diabetic nephropathy [14]. For example, miR-29c regulated the expression of inflammatory cytokines during the progression of diabetic nephropathy by regulating the expression of tristetraprolin [15]. Furthermore, in mice, miR-337 led to renal podocyte injury, with increased expression of inflammatory cytokines such as IL-6 and IL-18 [16]. Studies have shown that overexpression of miR-29b reduces glomerular injury and renal fibrosis by inhibiting macrophage-mediated podocyte inflammation [17]. However, whether miR-29b plays a role in diabetic nephropathy by regulating the inflammatory response of podocytes needs to be further explored.

PRKAB2 is a gene that codes the non-catalytic subunit beta 2 of the AMP-activated protein kinase (AMPK), a kinase involved in glucose metabolism and cell injury [18]. Research suggests that PRKAB2 may play an important role in diabetes [19]. The role of PRKAB2 in diabetic nephropathy is not fully understood and further studies are needed to uncover its mechanism of action and potential therapeutic applications.

In this study, we used high glucose (HG)-induced podocytes to mimic an *in vitro* cellular model of diabetic nephropathy. The effects of miR-29b and PRKAB2 on inflammatory response and podocyte apoptosis during the progression of diabetic nephropathy were also investigated in an *in vitro* model. Our study may provide new potential targets for the treatment of diabetic nephropathy.

## 2 MATERIALS AND METHODS

### 2.1 Experimental Workflow

The experimental flowchart is shown in Fig. (**1**). Firstly, the effects of different concentrations of glucose on apoptosis and inflammation of CIHP-1 cells were detected by flow cytometry and ELISA. miRNAs are filtered through Genecard and g: profiler databases. The effects of transfection with miR-inhibitor or miR-mimic on apoptosis and inflammation of HG-treated CIHP-1 cells were detected by flow cytometry and ELISA. Then, ENCORI, miRWalk, miRDB, and FunRich were used to screen downstream target genes of miR-29b, and the targeting relationship between miR-29b and PRKAB2 was verified by double luciferase assay and WB assay. Finally, rescue experiments verified the effects of miR-29b on apoptosis and inflammation of high-glucose-treated CIHP-1 cells by targeting and negatively regulating PRKAB2.

### 2.2 Cell Culture and Treatment

Culture and treatment of cells regarding previous studies [20]. The human podocyte cell line CIHP-1 cells were obtained from the Ximbio (London, UK) and cultured in DMEM culture medium (Procell Bio, Wuhan, China) supplied with 10% fetal bovine serum (FBS, SBJbio Life Sciences, Nanjing, China). For the HG-induced cell injury, cells were treated with 0-, 1-, 5-, and 25-mM glucose for 24 hours, among which the concentration of 25 mM was chosen for further mechanism study.

### 2.3 Flow Cytometry

Flow cytometry was used to evaluate the apoptosis rate in CIHP-1 cells [21]. Briefly, the podocytes were collected after the indicated treatment by using EDTA-free trypsin. The collected cells were centrifuged at 1000 rpm/min for 10 min. After washing the podocytes with sterile phosphate buffered saline, 500 μl binding buffer was added to the cells to be resuspended in single cells. Then, Annexin V-FITC (5 μl) and PI solution (5 μl) were added successively to the cell’s mixture. The mixture was placed in the dark at room temperature for 15 min, and then the cell apoptosis rate was examined by flow cytometry on a FACSCalibur (BD, Biosciences, USA).

### 2.4 Enzyme-linked Immunosorbent Assay (ELISA)

ELISA measured the inflammatory cytokines released by the CIHP-1 cells. Human IL-6 and IL-1β ELISA detection kits were obtained from Saipei Biotechnology (Wuhan, China). The TNF-α ELISA kit was obtained from Invitrogen (NY, USA). The MCP-1 ELISA kit was purchased from Abcam (Cambridge, UK). The inflammatory cytokines released by podocytes in the culture medium were determined under the protocols of manufacturers.

### 2.5 RNA Extraction and Real-time PCR

Trizol reagent (Invitrogen, NY, USA) was used to extract the total RNA of CIHP-1 cells following manufacturers' protocols [21]. After quantifying and purifying the extracted RNAs, 5 μg of total RNA were reversed and transcribed into the cDNA. Then, the single cDNA was performed by RT-PCR as the template by using the SYBR Taq (TaKaRa, Tokyo, Japan) under the procedure of 95°C for 6 min, and 40 circles of the process of 95°C for 60 s, 58°C for 30 s, 72°C for the 60s, followed with 72°C for 10 min. U6 and GAPDH were used as the internal control for miRNA expression and mRNA expression, respectively. The gene expression level was calculated by using the 2^−ΔΔCT^ method. The primers used in the present study were designed and synthesized by Genescript (Nanjing, China). The sequences of the primers are shown in Table **1**.

### 2.6 Cell Transfection

Cell transfection was performed with reference to previous studies [21], CIHP-1 cells were seeded in a 12-well plate (1×10^5^/well). After reaching 80%~90% confluence, the cells were transfected with miR-29b mimics or miR-29b inhibitor (50 pmol/mL) or PRKAB2 siRNA (40 pmol/mL) with indicated negative control by using Lipofectamine 3000 reagent (Invitrogen, NY, USA) according to the manufacturer’s instructions. MiR-29b mimics and inhibitors and the relative controls were purchased from Ribo Bio (Guangzhou, China). PRKAB2 siRNA was synthesized by Genescript (Nanjing, China). The sequence of the siRNA was as follows: si-PRKAB2: 5’-GGAGCACCAAGATTCCACTGA-3’; si-NC: 5’-GCTAGAATTGCGGTATTGACA-3’.

### 2.7 Western Blot Analysis

The total proteins were extracted from CIHP-1 cells by RIPA lysis and extraction buffer (Merck Life Sciences, Darmstadt, Germany) containing protease and phosphatase inhibitors (Sigma-Aldrich, Shanghai, China) [21]. The extracted proteins' concentration was determined using a commercialized Bradford protein assay kit (BOSTER Biotechnology, Wuhan, China). The exact amounts of protein (35 μg) were separated by electrophoresing on 12% SDS-PAGE, and the protein in the gels was then transferred to methanol methanol-pretreated polyvinylidene fluoride membranes (Millipore, Billerica, USA). The membrane with separated proteins on it was then submerged in a 5% skim milk solution at 25°C for 90 min, and the membrane was then immersed in indicated diluted primary antibodies at 4°C for at least 12 hours, followed by incubating with the indicated HRP-conjugated goat-anti-rabbit IgG secondary antibodies for 90 min at room temperature. The primary antibody against PRKAB2 was purchased from Abcam (Cambridge, UK). β-actin (Cambridge, UK) was used as the internal control. All the primary antibodies were diluted at the ratio of 1:1000. The protein bands were observed using the chemiluminescence under the enhanced ECL immunoblotting system (Tanon, Shanghai, China) and analyzed using ImageJ software (ImageJ 1.5, NIH, USA).

### 2.8 Bioinformatics Analysis

Diabetic nephropathy-related miRNAs were searched in Genecard (https://www.genecards.org/) and analyzed using g: profiler. Then, the predicted miRNAs expression in the control CIHP cells and the HG-induced CIHP cells was measured using qRT-PCR. The potential targets of miR-29b were predicted by the intersection of ENCORI, miRWalk, and miRDB database. Then, the potential target genes were subjected to FunRich (http://www.funrich.org/) to annotate the function, among which the immune system-related gene was chosen.

### 2.9 Luciferase Reporter Assay

The luciferase reporter assay verified the binding of miR-29b to PRKAB2 3’ UTR [21]. In detail, the pMIR-GLO report luciferase vector was obtained from Genepharma (Shanghai, China). The wild-type (WT) seed sequence of miR-29b in the 3’ UTR of PRKAB2 and the mutant type (Mut) of the seed sequence were amplified and inserted into the pMIR-GLO vector to construct the PRKAB2-WT and PRKAB2-Mut luciferase plasmid. The pMIR-GLO-PRKAB2 WT or pMIR-GLO-PRKAB2 Mut were co-transfected miR-29b mimic, or the control mimic, to CIHP cells for 48 hours. Then, the luciferase activity was determined using a commercialized luciferase assay kit (Promega, WI, USA) and normalized to the Ranilla luciferase activity.

### 2.10 Statistical Analysis

In all experiments, data were collected three times independently, and all data were tested for normality using the Shapiro-Wilk test, which is expressed as the mean ± Standard Deviation (SD). The statistical analysis was carried out by using GraphPad Prism5 software. Student's t-tests were used to compare the two groups, and one-way ANOVAs followed by Tukey's post hoc test were used when there were more than two groups to compare. *P* value < 0.05 was defined as statistically significant.

## 3 RESULTS

### 3.1 High Glucose Induced Inflammation and Cell Apoptosis in Podocytes

**Fig. (2):**
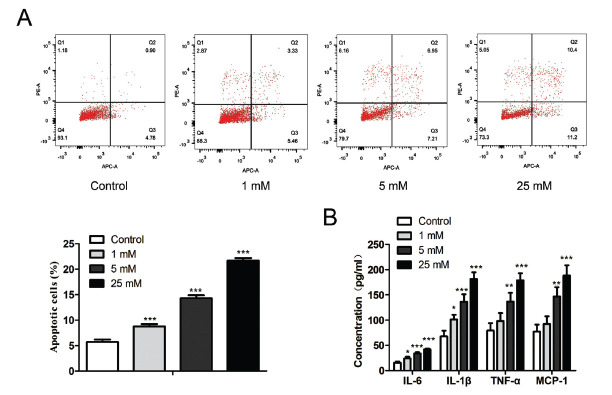
Effects of high glucose on podocyte inflammation and apoptosis. The podocytes were treated with 0, 1, 5, and 25 mM glucose for 24 hours. (**A**) Cell apoptosis was determined by using flow cytometry. (**B**) ELISA showed HG-induced release of IL-6, MCP-1, IL-1β, and TNF-α. The data are obtained from three replicated experiments and shown as the mean ± SD. *P<0.05, **P<0.01, ***P<0.001 *vs.* Control group. The Shapiro-Wilk test was used to analyze the normality of the data. Differences were analyzed using one-way ANOVAs followed by Tukey's post hoc test.

We first explored the effect of HG on podocytes. We treated the podocytes with glucose at the concentrations of 0, 1, 5, and 25 mM for 24 hours. We subsequently examined cell apoptosis and the inflammatory response in podocytes. Flow cytometry revealed a significant increase (F (3, 8) = 570.4, *P* < 0.0001) in the apoptosis levels of podocytes treated with 1 mM, 5 mM, and 25 mM glucose compared with the control group (Fig. **2A**). Additionally, ELISA results showed that HG induced an increased release of IL-6 (F (3, 8) = 61.07, *P* < 0.0001), IL-1β (F (3, 8) = 47.19, *P* < 0.0001), TNF-α (F (3, 8) = 24.25, *P* = 0.0002), and MCP-1 (F (3, 8) = 26.06, *P* = 0.0002) in a dose-dependent manner, thereby inducing an inflammatory response in podocytes (Fig. **2B**).

### 3.2 High Glucose Down-regulated miR-29b Expression in Podocytes

**Fig. (3):**
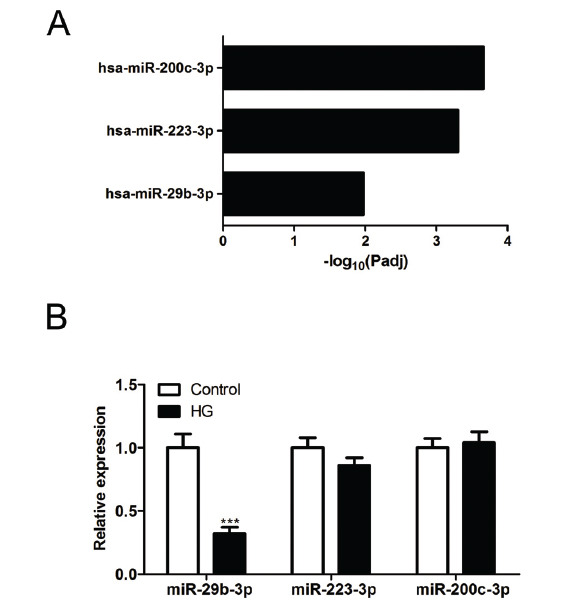
Effect of high glucose on the expression level of miR-29b in podocytes. (**A**) The diabetic nephropathy-related miRNAs were screened using Genecard (https://www.genecards.org/) and g: profiler. (**B**) The CIHP-1 cells were treated with 0 or 25 mM glucose for 24 hours. In HG-induced podocytes, miR-29b, miR-223-3p, and miR-200b-3p levels were determined by qRT-PCR. The data are obtained from three replicated experiments and shown as the mean ± SD. ***P<0.001 *vs.* Control group. The Shapiro-Wilk test was used to analyze the normality of the data. Differences were analyzed using student's t-tests.

MicroRNA has been reported to participate in the pathogenesis of diabetic nephropathy [22, 23]. We then screened the diabetic nephropathy-related miRNAs using Genecard (https://www.genecards.org/) and g: profiler, and found that miR-223-3p, miR-200b-3p and miR-29b may possess great functions in the development of diabetic nephropathy (Fig. **3A**). In HG-induced podocytes, miR-29b (t (4) = 9.827, *P* = 0.0006) was downregulated, compared to the control podocytes, as indicated by qRT-PCR (Fig. **3B**). The difference of miR-223-3p (t (4) = 2.414, *P* = 0.0732) or miR-200b-3p (t (4) = 0.6238, *P* = 0.5665) was not significant between the control cells and HG-induced cells (Fig. **3B**).

### 3.3 MiR-29b Knockdown Promoted Cell Apoptosis and Inflammatory Response in Podocytes

**Fig. (4):**
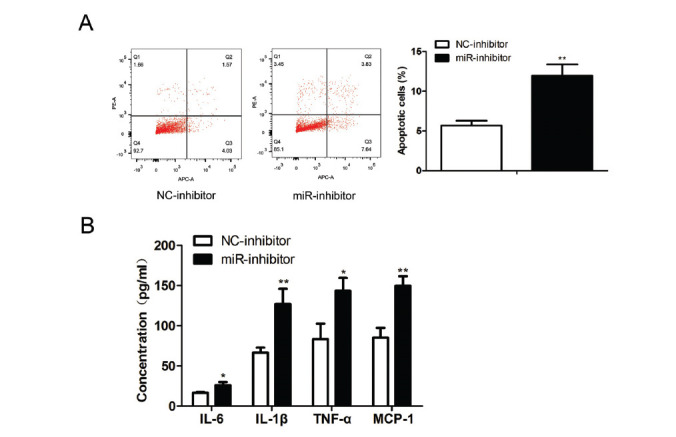
Effect of down-regulating miR-29b on podocyte apoptosis and inflammatory response. The podocytes were treated with miR-29b inhibitors (50 pmol/mL) and the relative negative controls for 48 hours. (**A**) Cell apoptosis of CIHP-1 cells was rate detected by using flow cytometry. (**B**) ELISA showed podocytes release of IL-6, MCP-1, IL-1β, and TNF-α. The data are obtained from three replicated experiments and shown as the mean ± SD. *P<0.05, **P<0.01 *vs.* NC-inhibitor. The Shapiro-Wilk test was used to analyze the normality of the data. Differences were analyzed using student's t-tests.

We then transfected podocytes with miR-29b inhibitors to downregulate miR-29b in podocytes. Flow cytometry revealed that the apoptosis rate was higher (t (4) = 7.069, *P* = 0.0021) in the cells transfected with miR-29b inhibitor than in the cells treated with control inhibitors (Fig. **4A**). In addition, the inflammatory cytokines IL-6 (t (4) = 3.955, *P* = 0.0168), IL-1β (t (4) = 5.354, *P* = 0.0059), TNF-α (t (4) = 4.151, *P* = 0.0143), and MCP-1 (t (4) = 6.635, *P* = 0.0027) released by miR-29b inhibitors-treated podocytes showed a higher level than that in the control cells, as revealed by ELISA (Fig. **4B**).

### 3.4 MiR-29b Overexpression Repressed HG-induced Cell Apoptosis and Inflammatory Response in CIHP-1 Cells

**Fig. (5):**
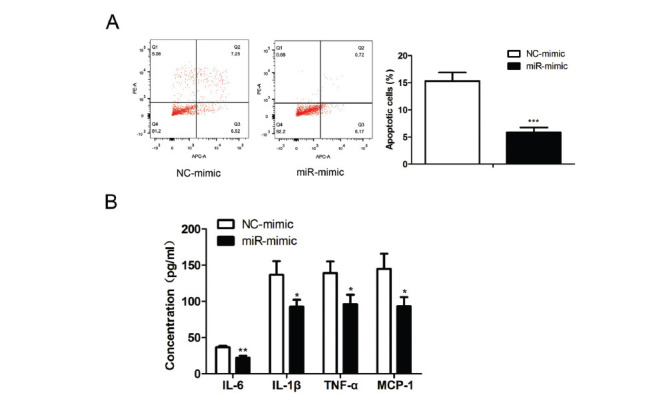
Effects of overexpression of MiR-29b on HG-induced podocyte apoptosis and inflammatory response. The CIHP-1 cells were stimulated with glucose at the concentration of 25 mM with miR-29b mimics (50 pmol/mL) and the relative negative controls for 48 hours. (**A**) Cell apoptosis rate was detected by using flow cytometry. (**B**) ELISA showed podocytes release of IL-6, MCP-1, IL-1β, and TNF-α. The data are obtained from three replicated experiments and shown as the mean ± SD. *P<0.05, **P<0.01, ***P<0.001 *vs.* NC-mimic. The Shapiro-Wilk test was used to analyze the normality of the data. Differences were analyzed using student's t-tests.

To further investigate the role of miR-29b in the podocyte injury in podocytes induced by HG, we transfected the HG-treated CIHP-1 cells with miR-29b mimics. We found that miR-29b mimic inhibited (t (4) = 8.927, *P* = 0.0009) cell apoptosis in podocytes (Fig. **5A**) compared with control group. In addition, podocytes treated with miR-29b mimics released less inflammatory cytokines IL-6 (t (4) = 6.814, *P* = 0.0024), IL-1β (t (4) = 3.645, *P* = 0.0219), TNF-α (t (4) = 3.589, *P* = 0.0230), and MCP-1 (t (4) = 3.660, *P* = 0.0216) than the control cells (Fig. **5B**).

### 3.5 MiR-29b Negatively Regulates PRKAB2 Expression

**Fig. (6):**
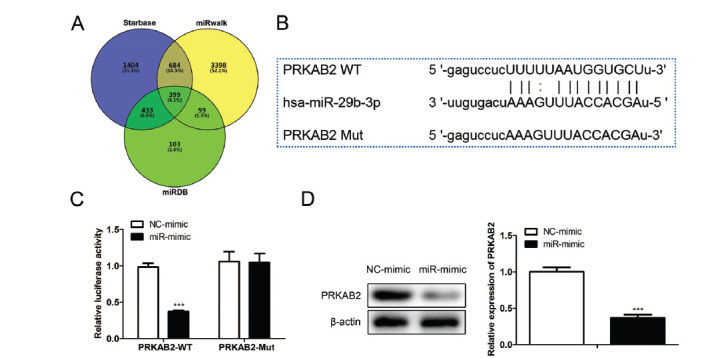
Relationship between miR-29b and PRKAB2. (**A**) Potential targets of miR-29b predicted by ENCORI, miRWalk, and miRDB database, and the intersection was shown by Venn diagram. (**B**) Potential binding sites of miR-129b within the region of PRKAB2 3’UTR. (**C**) The podocytes were performed transfection with a luciferase reporter plasmid combined with the miR-29b mimics, or the NC mimics for 48 hours, then the luciferase activity was determined. (**D**) PRKAB2 expression in CIHP cells was detected by using a western blot. The data are obtained from three replicated experiments and shown as the mean ± SD. ***P<0.001 *vs.* NC-mimic. The Shapiro-Wilk test was used to analyze the normality of the data. Differences were analyzed using student's t-tests.

We then investigated the underlying regulatory mechanism of miR-29b on HG-induced cell injury. By using ENCORI, miRWalk, and the miRDB database, we found 399 potential targets of miR-29b (Fig. **6A**). Using Funrich (http://www.funrich.org/) to perform functional enrichment analysis of the potential targets, we found that PRKAB2 was closely related to the immune system function, indicating PRKAB2 may be involved in the regulatory process in diabetic nephropathy. The potential binding site between miR-29b and PRKAB2 3’UTR is shown in Fig. (**6B**). We then performed a luciferase reporter assay to verify whether miR-29b directly bonded to 3’UTR of PRKAB2. As shown in Fig. (**6C**), miR-29b mimics decreased the luciferase activity significantly (t (4) = 19.98, *P* < 0.0001) in CIHP-1 cells co-transfected with the wild type of PRKAB2, which showed no influence (t (4) = 0.1216, *P* = 0.909) in CIHP-1 cells co-transfected with the mutant type of PRKAB2, indicating that miR-29b bound to the 3’UTR of PRKAB2. We further confirmed that miR-29b inhibited (t (4) = 14.55, *P* = 0.0001) PRKAB2 protein expression in CIHP-1 cells (Fig. **6D**).

### 3.6 MiR-29b Downregulation Promoted Inflammation and Cell Apoptosis in Podocytes through Upregulation PRKAB2

**Fig. (7):**
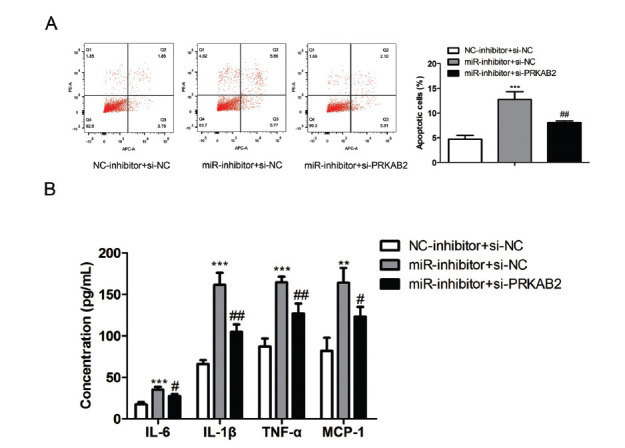
Down-regulated miR-29b regulates the effects of PRKAB2 on podocyte inflammatory response and apoptosis. The CIHP-1 cells were transfected with miR-29b inhibitors (50 pmol/mL), with or without PRKAB2 siRNA (40 pmol/mL) for 48 hours. (**A**) The cell apoptosis rate of the CIHP cells was detected by using flow cytometry. (**B**) ELISA showed podocytes release of IL-6, MCP-1, IL-1β, and TNF-α. The data are obtained from three replicated experiments and shown as the mean ± SD. **P<0.01, ***P<0.001 *vs.* NC-inhibitor+si-NC. ^#^*P*<0.05, ^##^*P*<0.01 *vs.* miR-inhibitor+si-NC. The Shapiro-Wilk test was used to analyze the normality of the data. Differences were analyzed using one-way ANOVAs followed by Tukey's post hoc test.

To further verify the effect of the miR-29b/PRKAB2 axis in regulating cell apoptosis and inflammation, we treated the cells with miR-29b inhibitors, with or without knockdown of PRKAB2. Flow cytometry revealed that the knockdown of PRKAB2 inhibited the increased (F (2, 6) = 45.35, *P* = 0.0002) cell apoptosis induced by miR-29 inhibitors (Fig. **7A**). Furthermore, ELISA showed that miR-29b inhibitors enhanced the release of inflammatory cytokines IL-6 (F (2, 6) = 29.7, P=0.0008), IL-1β (F (2, 6) = 64.38, *P* < 0.0001), TNF-α (F (2, 6) = 47.58, *P* = 0.0002), and MCP-1 (F (2, 6) = 21.43, *P* = 0.0019), while knockdown of PRKAB2 abolished the effect of miR-29b inhibitors (Fig. **7B**).

## 4 DISCUSSION

Diabetic nephropathy, one of the most severe complications of diabetes, diabetic nephropathy exhibits a high incidence, accounting for almost one-third of type I and type II diabetes mellitus patients [24]. Furthermore, diabetic nephropathy stands as the primary cause of End-Stage Renal Disease (ESRD), often necessitating renal replacement therapy [24]. Diabetic nephropathy is characterized by the thickening of the glomerular basement membrane, the expansion of the glomerular interstitial and interstitial matrix, and the loss of podocytes, which lead to the occurrence of microalbuminuria and the decline of renal function [25]. However, the exact pathological mechanism of diabetic nephropathy remains not fully understood. Although hyperglycemia is considered the main driving force for the development of diabetic nephropathy, there is considerable uncertainty about the underlying mechanism of chronic hyperglycemia and other metabolic features of diabetes leading to diabetic nephropathy.

The injury and apoptosis of podocytes are central factors in the progression of diabetic nephropathy [26]. As part of the glomerular filtration barrier, podocytes prevent plasma protein from escaping from the glomerular circulation by forming a filter gap structure [27]. Podocyte injury results in progressive proteinuria [28]. Hyperglycemia can trigger cell injury, apoptosis, and inflammation in podocytes, serving as the initiating factors in the development of diabetic nephropathy [29]. In our present study, we conducted *in vitro* experiments on podocytes exposed to varying glucose concentrations to replicate hyperglycemic conditions. We found that high glucose stimulation induced increased cell apoptosis and inflammatory response in a dose-dependent manner.

MicroRNAs are a type of small non-coding single-stranded RNAs that could directly regulate the protein expression of almost 60% of human genes [30]. Many miRNAs function with a protective or destructive effect on diabetic nephropathy. For example, miR-342 was reported to inhibit renal interstitial fibrosis and renal mesangial cell apoptosis in mice with diabetic nephropathy by downregulating the expression of SRY-box 6 (SOX6) [31]. MiR-320a was reported to induce diabetic nephropathy via inhibiting MafB [32]. MiR-29b-3p suppresses the progression Of diabetic nephropathy (DN) in mice by inhibiting the expression of Enhancer of Zeste Homolog 2 (EZH2) [33]. The study of “exploring miR-29b-3p and other miRNAs for early diagnosis, monitoring and treatment of DN” found that the decreased expression of miR-21-3p and miR-192-5p was associated with the occurrence and development of DN, which may be potential biomarkers for early diagnosis of DN [34], and the review of recent research advances revealed that miR-126, miR-29b and miR-125a have been implicated in diabetes-induced microvascular complications, whereas miR-146a was found to be associated with all of these complications [35]. MiR-29b was significantly downregulated in the mice with type II diabetes, and miR-29b regulated diabetic aortic remodeling [36]. In HG-induced Müller cells, miR-29b decreased significantly, and upregulation of miR-29b prevented HG-induced cell injury [37]. MiR-29b mimics suppressed macrophage-mediated inflammation in podocytes and attenuated HDAC4 expression, glomerular damage, and renal fibrosis [17]. MiR-29b-3p is inhibited in LPS-induced acute kidney injury (AKI). In LPS-induced AKI, down-regulation of miR-29b-3p exacerbates podocyte injury by targeting HDAC4 [38]. Based on these previous studies, in this study, we found that HG-induced a noticeable decrease of miR-29b expression in podocytes. In addition, we found that inhibition of miR-29b promoted the cell apoptosis and inflammatory response in podocytes, and miR-29b overexpression prevented HG-induced cell injury and inflammatory response, indicating that miR-29b possessed a crucial regulatory role in podocyte injury induced by HG.

MiRNAs play various critical physiological roles by targeting gene translation through their binding to the 3’UTR of genes [39]. Through bioinformatics predictions, we identified PRKAB2 as a potential target of miR-29b. Subsequent research, utilizing a luciferase reporter assay, confirmed the direct binding of miR-29b to the 3’UTR of PRKAB2. Western blot and qPCR further confirmed that miR-29b downregulated PRKAB2 expression. PRKAB2 is a gene that codes for the non-catalytic subunit beta 2 of the AMP-activated protein kinase (AMPK), a kinase involved in glucose metabolism and cell injury. This research found that PRKAB2 knockdown abolished the increased cell apoptosis and inflammatory response induced by miR-29 inhibitors, indicating that miR-29b protected podocytes from HG-induced cell apoptosis and inflammation through negatively regulating PRKAB2.

In addition, miR-29b was also found to alleviate the inflammatory response after ischemic stroke through tumor necrosis factor (C1QTNF) 6 [40] While targeting the miR-29b/C1QTNF6 axis in cerebral ischemic injury promotes leukocyte inflammation [41], miR-29b could also reduce alcohol-induced inflammation by targeting the signal transducer and activator of transcription 3 (STAT3) [42]. In this study, we identified that miR-29b possessed a vitally important function in regulating inflammation and cell apoptosis in HG-treated CIHP cells through regulating PRKAB2 expression. Thus, our research provides a new target and insights into the molecular mechanism of diabetic nephropathy.

However, there are some limitations to this study. In this study, only *in vitro* validation experiments were carried out, and *in vivo* experiments were lacking. In addition, the lack of clinical data is one of the limitations of this study. Therefore, it is highly necessary to validate the role of miR-29b in diabetic nephropathy through *in vivo* and clinical data in the future. In addition, studies on the downstream signaling pathways of PRKAB2 are also necessary to further explore the molecular mechanism of diabetic nephropathy.

## CONCLUSION

In the present study, high glucose treatment decreased the expression level of miR-29b-3p in CIHP cells, and increased the level of apoptosis and inflammation. MiR-29b has been found to protect podocytes from high glucose-induced inflammation by negatively regulating PRKAB2, providing new insights into the molecular mechanisms by which miR-29b regulates inflammation and the pathological processes involved.

## Figures and Tables

**Fig. (1):**
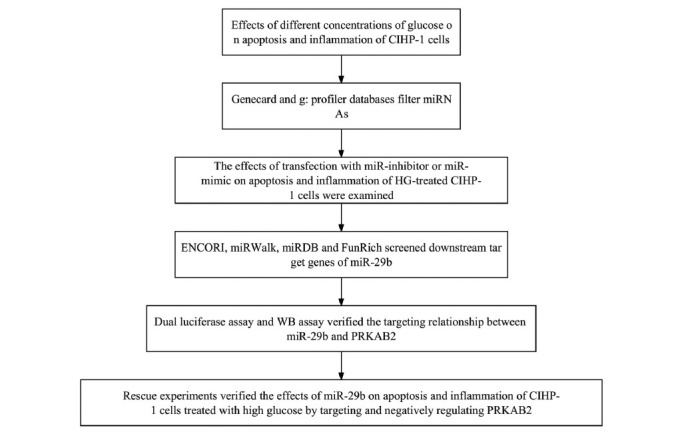
Flow diagram of the experiment.

**Table 1:** Primer sequences of genes in RT-qPCR assay.

**Gene**	**Forward (5'-3')**	**Reversed (5'-3')**
MiR-29b	CGTAGCACCATTTGAAATCAGTGTT	GTGCAGGGTCCGAGGT
PRKAB2	AGGATTTGGAGGACTCCGTA	TCAGTGGAATCTTGGTGCTC
U6	AACGCTTCACGAATTTGCGT	AACGCTTCACGAATTTGCGT
GAPDH	ACCCAGAAGACTGTGGATGG	CACATTGGGGGTAGGAACAC

## Data Availability

All data generated or analyzed during this study are available from the corresponding author.

## LIST OF ABBREVIATIONS

3' UTR: 3' Untranslated Region
AMPK: AMP-activated Protein Kinase
ESRD: End-stage Renal Disease
SD: Standard Deviation
WT: Wild-type
